# Data on cell spread area and directional contraction in human umbilical vein endothelial cells on fibronectin and on collagen type I-coated micro-posts

**DOI:** 10.1016/j.dib.2016.01.033

**Published:** 2016-01-29

**Authors:** Jing-Jing Han, Hui-Foon Tan, Chen Feng, Wei-Kiat Wee, Shang-You Tee, Suet-Mien Tan

**Affiliations:** a School of Physical and Mathematical Sciences, Nanyang Technological University, 21 Nanyang Link, 637371, Singapore; b School of Biological Sciences, Nanyang Technological University, 60 Nanyang Drive, 637551, Singapore

## Abstract

Fibronectin and collagen type I are abundant extracellular matrix proteins that modulate cell mechanics and they regulate angiogenic sprouting. In this data article, fibronectin- or collagen type I-coated micro-posts were used to examine the traction force, cell spread area and directional contraction of human umbilical vein endothelial cells (HUVECs).

## Specifications Table

**Table:** 

Subject area	*Biology.*
More specific subject area	*Cell-extracellular matrix adhesion and contraction force.*
Type of data	*Images and graphs.*
How data was acquired	*Time-lapse images of HUVECs on ECM-coated micro-posts were captured on an Olympus IX70, 40X/0.60 Ph2 objective, equipped with CCD camera and housed in a custom-built plastic box connected to a temperature controlled 37 °C and CO_2_ incubator. Immunofluorescent images were acquired on a confocal laser scanning microscope Zeiss LSM510, Carl Zeiss.*
Data format	*Analyzed.*
Experimental factors	*HUVECs, fibronectin and collagen type I were used.*
Experimental features	*Comparative analyses of cell traction forces of HUVECs on fibronectin- or collagen type I-coated micro-posts.*
Data source location	*Singapore, Singapore.*
Data accessibility	*Data are with this article.*

## Value of the data

• These data provide for the first time comparative analyses of contraction forces exerted by HUVECs on fibronectin or collagen type I-coated micro-posts.
• These data are useful for researchers in the field of cell adhesion and mechano-biology.
• These data are also useful to researchers in the field of wound healing.
• These data provide opportunities for collaborative studies to investigate the underlying mechanism by which differences in directional force contraction on different ECM proteins are generated.

## 1 Data

**Fig. 1:**
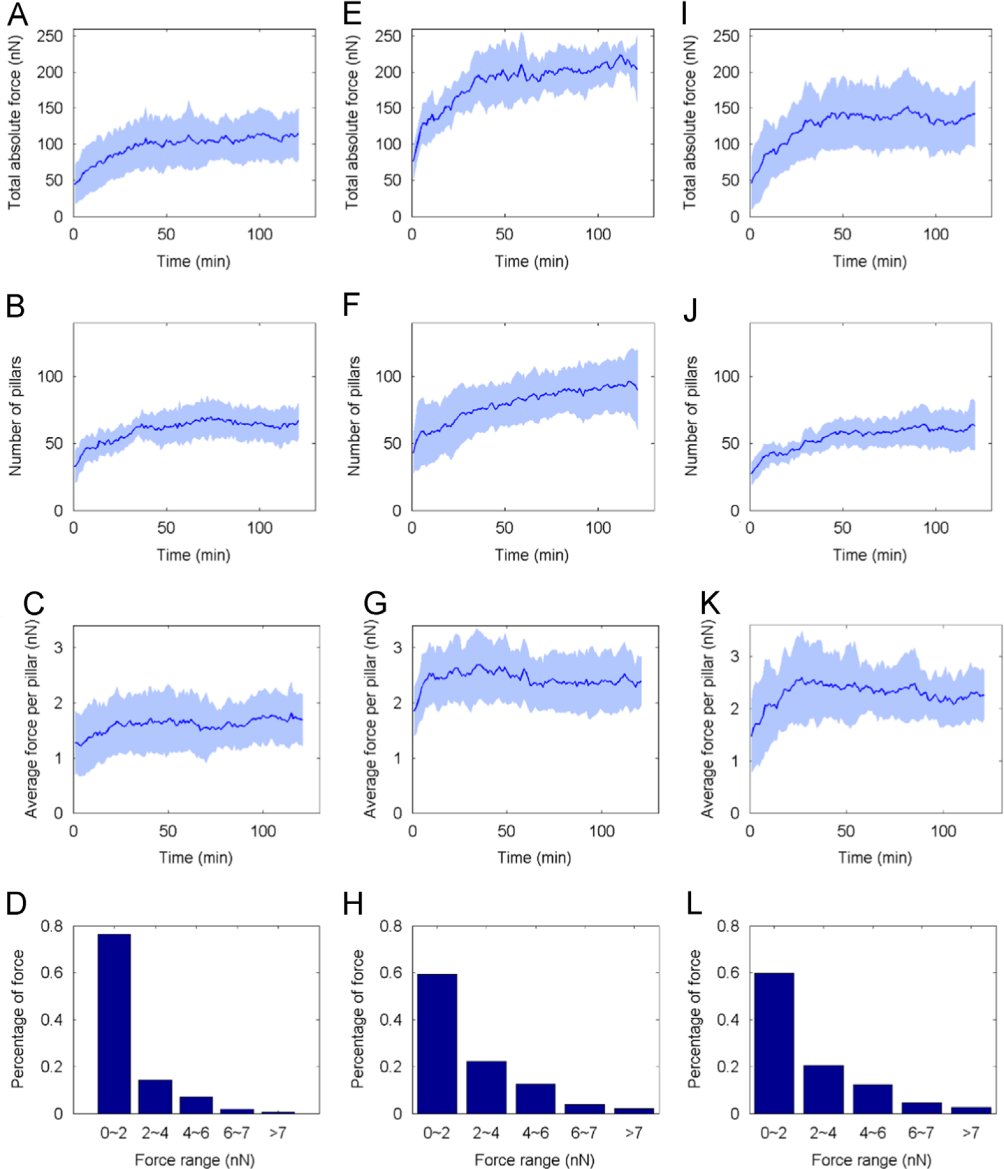
Force measurements of HUVECs on uncoated (A–D), fibronectin (E–H) or collagen type I (I–L)-coated micro-posts. (A, E, I) Plots of total absolute force (nN) against time (min) (duration=2 h) of cells on micro-posts. (B, F, J) Plots of number of pillars occupied per cell against time (min). (C, G, K) Plots of average force exerted on each pillar against time (min). (D, H, L) Normalized distribution of forces exerted on all the pillars for 2 h. (A, B, C, E, F, G, I, J, K), *n*=5 for uncoated pillars. *n*=7 for pillars coated with either fibronectin or collagen type I. Data are presented as mean (dark blue line)±SD (pale blue region). Major population of microposts in all groups experienced 0–2 nN of contraction force (D, H, L). Statistical analyses of these groups using unpaired Student׳s *t* test: FN vs Uncoated (*p*=0.0287); COL vs Uncoated (*p*=0.0264); FN vs COL (*p*=0.9116).

**Fig. 2:**
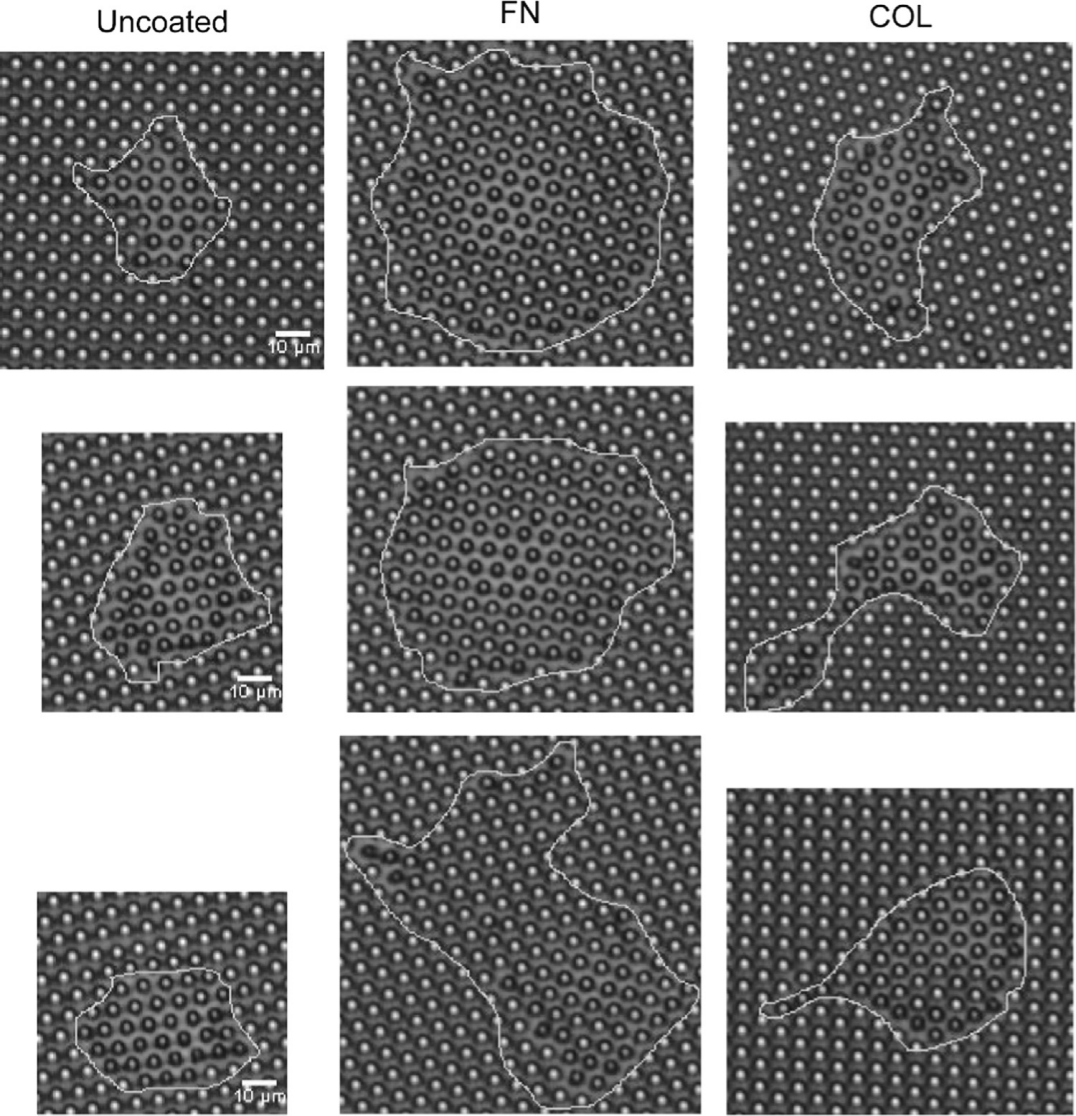
Representative still images of cells on uncoated, fibronectin (FN)-coated or collagen type I (COL)-coated micro-posts. Representative still images of cells at 2 h are shown. The cell outline was traced using the Image J software. All images are of the same magnification. Reference scale bar (10 μm) is shown in the left panel (uncoated group).

**Fig. 3:**
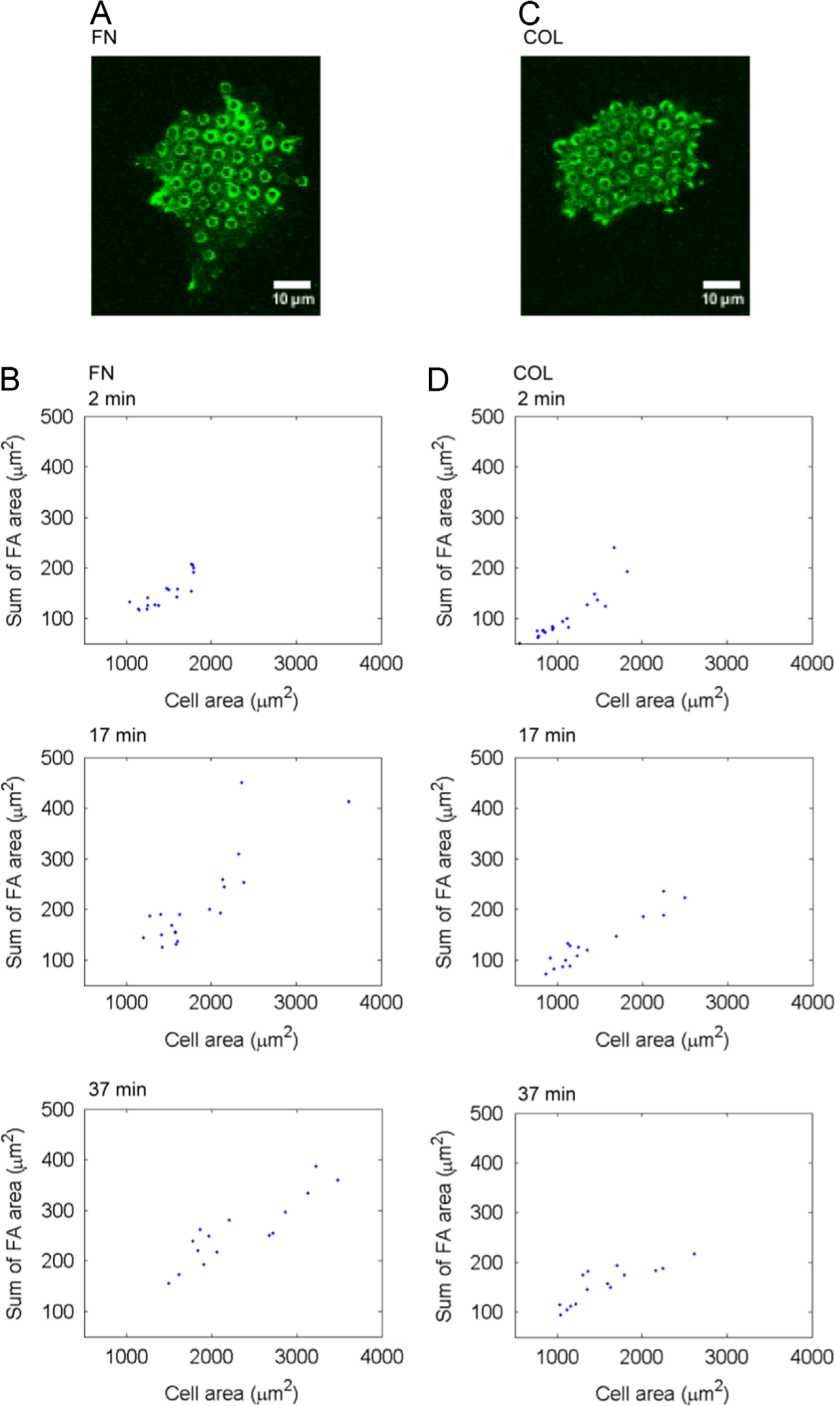
Measurements of focal adhesion area of HUVECs on micro-posts coated with either fibronectin (A, B) or collagen type I (C, D). (A, C) Representative immunofluorescence microscopy images of fixed cells on micro-posts stained for focal adhesion protein paxillin at the 17th min. (B,D) Plots of total focal adhesion area (μm^2^) per cell against cell spread area (μm^2^) at indicated time points. Each blue dot represents one cell.

**Fig. 4:**
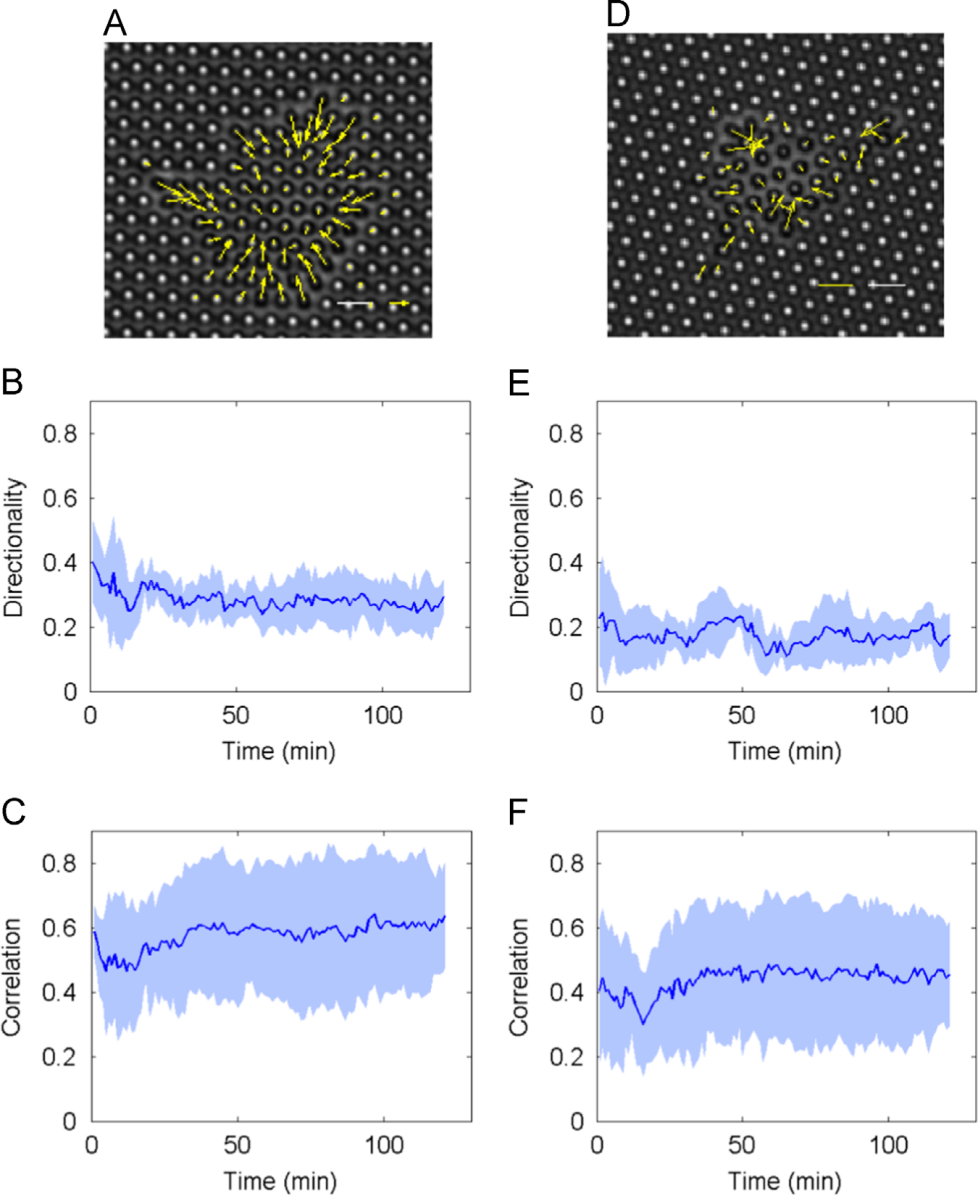
Measurements of directional contractions of HUVECs on micro-posts coated with either fibronectin (A, B, C) or collagen type I (D, E, F). (A, D) Representative force maps of cells on micro-posts at ~60 min. The yellow arrow indicates the force vector on each pillar. Scale bars: force magnitude 5 nN (yellow) and dimension 10 μm (white). (B, E) Plots of directional contractions against time (min). (C, F) Plots of the average correlation of force on all neighboring pillar pairs against time (min). (B, C, E, F), *n*=7 for each plot. Data shown are mean (dark blue line)±SD (pale blue region).

The data in this article compare the contraction force, cell spread area, focal adhesion area and directional contraction of HUVECs on microposts coated with either fibronectin or collagen type I. Data are presented in Fig. 1, Fig. 2, Fig. 3, Fig. 4.

## 2 Experimental design, materials and methods

### 2.1 Experimental design

HUVECs were settled on micro-posts coated with either fibronectin or collagen type I. Time-lapse live cell imaging was performed on a microscope equipped with CCD camera and a temperature and CO_2_ controlled chamber. Images acquired were analyzed to determine cell-spread area, cell-traction force, focal adhesion area and directional contraction.

### 2.2 Materials

HUVECs were purchased from a commercial source (Lonza, Basel, Switzerland) and cultured in endothelial cell growth medium containing supplements (Lonza) at 37 °C in a humidified 5% CO_2_ incubator.

The micro-posts were kind gifts from Prof. Jianping Fu (University of Michigan, U.S.A.). Microposts fabrication method has been reported [1]. The parameters of the microposts are: height (8.3 µm), diameter (1.83 μm), spacing between posts (6 µm), spring constant (7.22 nN/µm) and effective modulus (5.65 kPa). Microposts were coated with 20 µg/ml human plasma fibronectin (Sigma Aldrich, St Louis, MO) in PBS for 2 h at 37 °C. Micro-posts were coated with 20 µg/ml rat tail collagen type I (BD Bioscience) in 0.02 M acetic acid for 2 h at 37 °C. All microposts were washed in PBS before used.

Other materials used are described in the following section.

## 3 Methods

HUVECs (4×10^5^ cells) were settled onto ECM-coated micro-posts that were embedded on a cover-glass culture dish. Cells were allowed to settle for 10 min under culture conditions. The culture dish was then placed in an adapter (with 5% CO_2_ supply) on the stage of an inverted fluorescence microscope (Olympus IX70, 40X/0.60 Ph2 objective, equipped with a CCD camera) (Olympus, Tokyo, Japan) which was housed in a custom-built plastic box connected to a temperature controlled 37 °C incubator. Individual cell was chosen in a random manner within 3 min after placing the micro-post dish on the microscope stage. Any cell that was in contact with another cell was not chosen since cell–cell contact would interfere with the analyses of cell spreading and the defection of microposts. In addition, at any one time, only 1 isolated cell could be selected per experiment in order to achieve proper focusing on the microscope stage and to obtain suitable images for subsequent analyses. To obtain the next set of data, the microposts that were embedded on a cover-glass culture dish were cleaned by ultrasound in a waterbath sonicator (Elma, Schmidbauer GmbH, Germany). After which, the microposts were re-coated with the appropriate ECM protein. HUVECs were settled on the microposts aforementioned and the experiment was repeated. In Fig. 1, data on uncoated microposts were from five experiments (*n*=5) and data on fibronectin and collagen type I-coated microposts were each from seven experiments (*n*=7). Phase contrast images were captured every 1 min for 2 h and processed using the MetaMorph software.

Acquired images were exported as 16-bit TIFF images and the centroid of each pillar was identified with a custom 2D particle tracking program [2] (Mathworks, Novi, MI). The Top-ideal Method (T-I) [3] was used to calculate the pillar deflection. In brief, all the pillars are supposed to be spaced uniformly, then based on the positions of the undeflected pillars at the first frame, we estimate the original positions of the occupied pillars using two-dimensional linear interpolation to get the theoretical unoccupied frame (TUF). The deflection was determined by the difference between the pillar centroid in the TUF and that in the actually captured frame. The drift of microscope setup during experiment was calculated as the average vectorial displacement of the unoccupied pillars in each frame compared to the corresponding initial positions in TUF. After drift elimination, there was still small deflection for the unoccupied pillars. Based on our trial tests, 0.06–0.10 μm was chosen as a threshold. Only pillars whose deflection magnitudes were above the threshold were considered to be occupied by cell contraction. The traction force was obtained by multiplying the deflection with the nominal spring constant of pillar. The total absolute force exerted by each cell and the number of pillars under each cell were calculated and plotted against time. The number of pillars under each cell, which corresponds to the spread area of the cell, was determined by inspecting each cell-image captured over the 2 h duration. Values were plotted against time. The average force exerted on each pillar per cell was also calculated and plotted against time. Unpaired Student׳s *t* test was performed to determine the statistical significance of the difference between pillars that experienced contraction force in the range of 0–2 nN amongst different groups, namely FN vs Uncoated, COL vs Uncoated, and FN vs COL. Pillars in the force range of 0–2 nN were chosen because they formed the major population in the normalized distribution of force in all three groups of cells.

In experiments measuring focal adhesion area, HUVECs (4×10^5^) were also settled onto ECM-coated micro-posts as described above. Cells were fixed in 3.7% (w/v) paraformaldehyde in PBS for 10 min at R.T. and permeabilized in cytoskeleton stabilization buffer (100 mM, 300 mM sucrose, 3 mM MgCl_2_, 1 mM EGTA and 10 mM PIPES, pH 6.8) containing 0.3% (v/v) Triton X-100 for 1 min at R.T. [4]. Cells were washed once in PBS and incubated in PBS containing 1% (w/v) BSA for 30 min at R.T. Cells were then incubated in PBS containing 1% (w/v) BSA and 5 µg/ml anti-paxillin (clone 5H11) (Merck) at 4 °C overnight followed by Alexa Fluor® 488-conjugated goat anti-mouse IgG secondary antibody (1:600 dilution) (Invitrogen, Carlsbad, CA). Cells were examined under a confocal laser scanning microscope (Zeiss LSM510, Carl Zeiss, Germany) with 63× oil immersion objective lens. Data were analyzed using the Zen 2012 software. To quantify the focal adhesion size, fluorescent images were converted to black-and-white images using the ImageJ software (open source, imagej.net). Background subtraction was performed to reduce noise by setting threshold limit. The number of pixels with values above the threshold which represent the size of focal adhesion was calculated. To quantify the cell spread area, fluorescent images were converted to black-and-white images using the ImageJ software (open source, imagej.net), and Canny edge detection was applied to binarize the images (Mathworks, Novi, MI). Image dilation, erosion, and fill operations were used to fill in the gaps. The number of white pixels was summed to determine the cell spread area.

The directionality of contraction was determined by taking the magnitude of the sum of force vectors divided by the sum of their magnitudes [5]. A value of 1.0 suggests parallel forces whereas a value of 0 suggests balanced forces. The correlation value of force vectors of neighboring pillars by taking the cosine of the angle between the two neighboring force vectors. A correlation value of 1.0 is attained when the angle is 0 (i.e. when the two neighboring force vectors are parallel).

## Conflict of interests

The authors have no competing financial interests.
